# Supplementary material for: Epigenetic Mechanisms in Fabry Disease: A Thematic Analysis Linking Differential Methylation Profiles and Genetic Modifiers to Disease Phenotype
Source: Curr Issues Mol Biol. 2025 Oct 16;47(10):855. doi: 10.3390/cimb47100855 (PMC12564460; doi:10.3390/cimb47100855)

Supplementary Table S1: Scale for the Assessment of Narrative Review Articles (SANRA)

Item*	Score**	Reason for score
1, Justification of the review's importance	2	Introduction explained the importance of the work and the justification for undertaking the review.
2, Aims of the review	2	The research question and aim of the review was described in detail.
3, Description of the search	2	Methods include a description of the literature search with search terms and eligibility criteria. The literature search flow chart (Figure 1) is also provided.
4, Supporting references	2	Key information was supported by references from peer reviewed journals from experts in the field.
5, Scientific reasoning	2	Study characteristics were described and the scientific reasoning within the discussion included supporting literature evidence for DNA methylation and genetic modifiers in Fabry disease.
6, Presentation of data	2	Data were presented in Table 1 and key findings from each of the 20 eligible studies were summarized.
**Total**	**12**	

* Items were derived from Baethge C, Goldbeck-Wood S, Mertens S. SANRA-a scale for the quality assessment of narrative review articles. Res Integr Peer Rev. 2019 Mar 26;4:5 [40].
** Scores are rated from 0 (low quality) to 2 (high quality).

Supplementary Table S2: Thematic Analysis Framework and Identification of Themes

Statements

There was also no difference in leukocyte telomere length in patients receiving disease specific therapy and those not receiving.

The study showed the accumulative effects of *GLA* mutation and BDP methylation on disease severity

Inflammatory mediators are doubled in the plasma of Fabry patients treated with ERT.

The study concluded that somatic mosaicism could be an important disease modifier in Fabry disease.

In male patients, genetic and non-genetic factors might be associated with the clinical phenotype.

Variation in the mitochondrial genome could contribute to the pathogenesis of Fabry disease.

The calcitonin receptor methylation profile could indicate disease severity and potentially be an epigenetic biomarker in patients with ERT.

Several genes were found to be differentially methylated, and the methylation status of these genes could help to predict disease progression and organ involvement.

In 21 genes that had differential methylation profiles, *collagen type IV alpha 1 and alpha 2 genes* showed hypomethylation.

The genetic algorithm can be used to assess whether the variant in FD is pathogenic or likely pathogenic.

Overall, there was a clear correlation between FASTEX scores, sphingolipids accumulation and demethylation of the *GLA* gene.

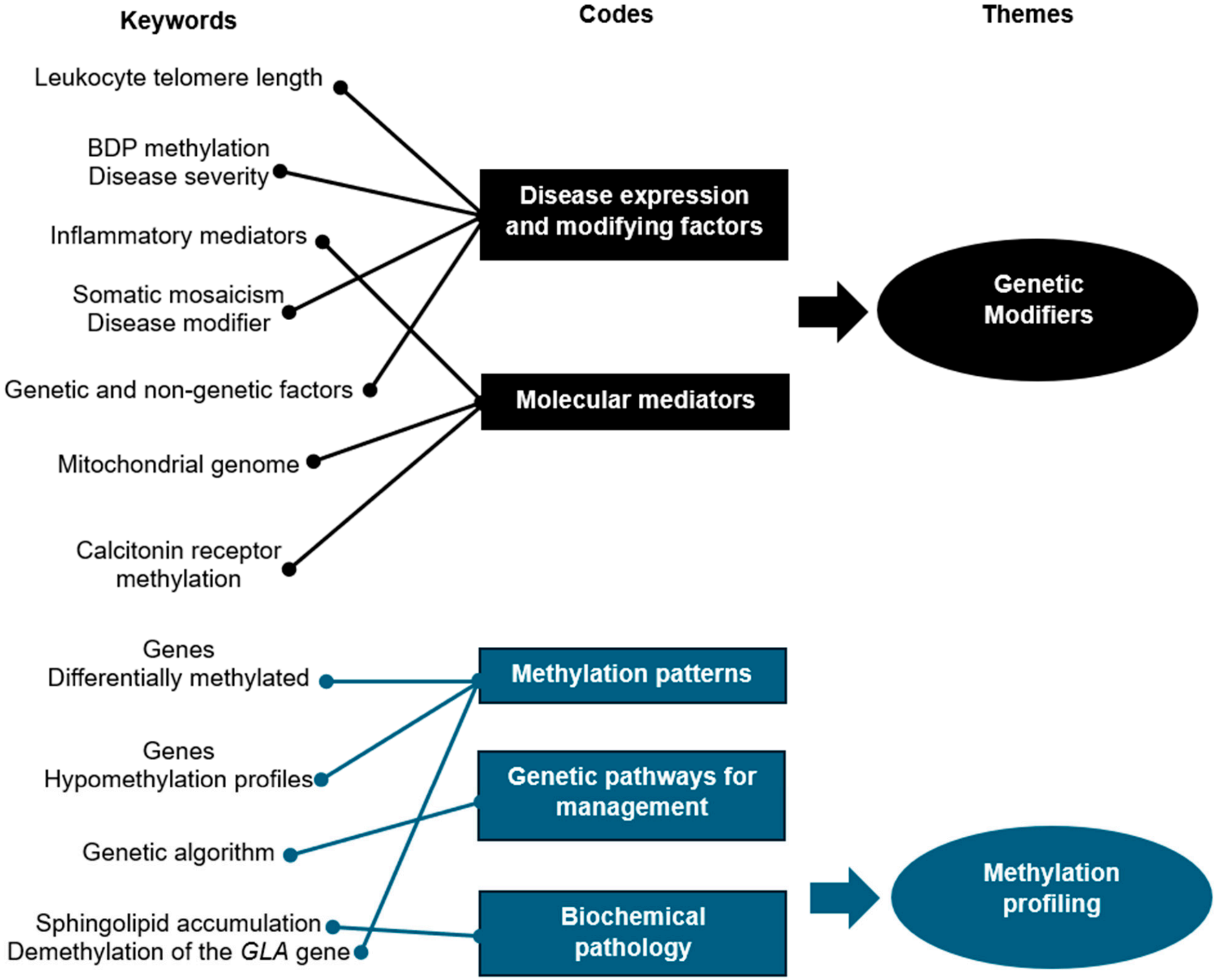

The study identified a novel mutation *c.270C>G* (p. Cys90Trp) in the *GLA* gene.

Overall, the study showed an association between clinical severity, dysregulated autophagy and methylation of wild alleles in Fabry disease.

Methylation of the CpG island in the *GAL* gene was reported.

No correlation was found between XCI and clinical severity scores.

In summary, the authors demonstrated that in Fabry disease, XCI patterns have limited use in understanding disease severity.

Better understanding of tissue-specific and age-related XCI patterns can help to minimise potential bias when interpreting XCI studies in Fabry disease.

The main findings from the study demonstrated that XCI skewing cannot explain the clinical severity in Fabry disease

The study demonstrated that clinical phenotype in Fabry disease patients s not influenced by XCI

Overall, the study suggested that XCI can impact the clinical phenotype in individuals with Fabry disease.

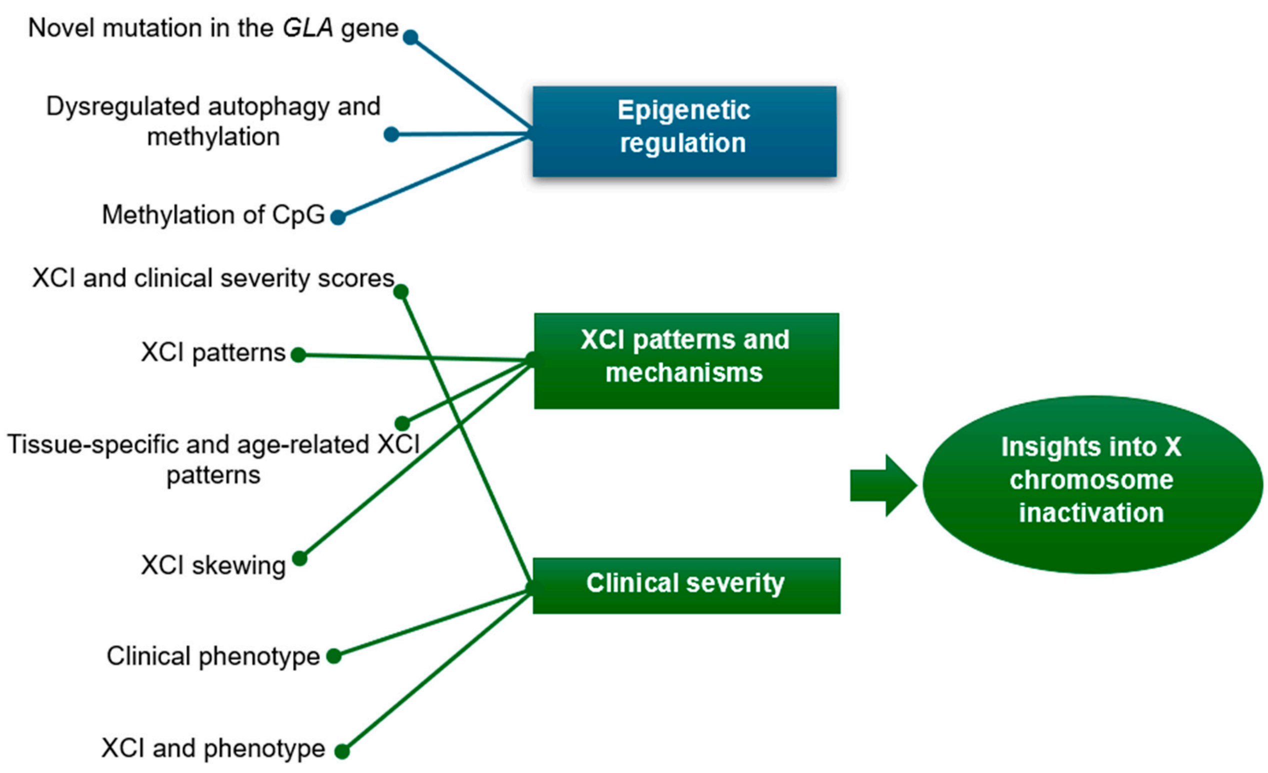

Supplement: Supplementary file 1 [file cimb-47-00855-s001.zip › cimb-3900561-Supplementary.pdf]
